# Variations in the Termination of the Popliteal Artery: A Multidetector Computed Tomography Angiography (CTA)-Based Retrospective Study

**DOI:** 10.7759/cureus.63092

**Published:** 2024-06-25

**Authors:** Anurag Rai, Jyoti Chopra, Amber Irfan, Shubhajeet Roy, Gourav Gourav, Anit Parihar, Shailendra Kumar

**Affiliations:** 1 Thoracic Surgery, King George's Medical University, Lucknow, IND; 2 Anatomy, King George's Medical University, Lucknow, IND; 3 Gandhi Memorial and Associated Hospitals, King George's Medical University, Lucknow, IND; 4 Interventional Radiology, King George's Medical University, Lucknow, IND

**Keywords:** computed tomography angiography, 3d reconstruction image, 128-slice ct scan, anatomical variations, popliteal artery

## Abstract

Background: Comprehension of the intrucate anatomy and variations in the termination of the popliteal artery (PA) is increasingly essential for endovascular interventionists, plastic surgeons, vascular surgeons, and orthopedic surgeons, due to the rise in procedures like embolectomy, vascular grafting, free fibular flap surgery, and high-tibial osteotomy. Few studies from India have reported on the variant anatomy of PA termination, and none have used 128-slice tomography. This study aimed to observe the terminal branching pattern of the PA and the morphology of its terminal branches using 128-slice computed tomography angiography (CTA) and to analyze its relation to gender and laterality.

Methodology: A retrospective review of CTA images of 181 lower extremities from 100 patients (137 males and 44 females), aged five to 75 years, was conducted.

Results: The usual type I-A pattern was found in 75.69% of cases, while 24.31% exhibited variant patterns. Type III was the most common variation observed (19.34%), with type III-A being the most prevalent (11.05%). Types II-B and II-C were not observed. Among 84 bilaterally examined cases, 19.05% had unilateral variations and 15.48% had bilateral variations, with 8.33% showing bilaterally similar variations and 7.14% dissimilar variations. No significant difference in branching patterns was found between genders or sides. The mean length of the tibial-peroneal trunk (TPT) in the type I-A pattern was 3.00 ± 0.99 cm (right side: 3.21 ± 1.02 cm; left side: 2.82 ± 0.93 cm; males: 2.9 ± 1.00 cm; females: 3.37 ± 0.85 cm), with statistically significant differences between sides and genders. In the type II-A pattern, the mean TPT length was 7.16 ± 3.75 cm. An exceptionally long TPT (12.97 cm) was noted in one case of the III-B pattern.

Conclusion: There is a high prevalence of variation in the termination pattern of the PA. Knowledge of these variations is crucial for any interventions in this region to avoid postoperative vascular complications and reduce patient suffering.

## Introduction

The superficial femoral artery continues as the popliteal artery (PA) and stretches from the adductor hiatus to the inferior border of the popliteus, where it bifurcates into the two terminal branches - the anterior tibial artery and tibioperoneal trunk. The tibioperoneal trunk then bifurcates into posterior tibial artery (PTA) and peroneal artery (PA) [1]. Normally, the PA, as per classical cadaveric descriptions, bifurcates at the lower border of the popliteus, but this cannot be ascertained in angiographic studies. Hence, the tibial plateau is considered the reference point [2].

Variations in the PA branching pattern are not uncommon; these variations were first categorized into various types in angiographic and post-mortem studies [3-5]. The basis of classification is the level at which the artery bifurcates, and it includes three primary types and their 10 subtypes, according to Kim et al.’s classification [4]. Later, Foul et al. proposed an additional variant type IV in cases where PrA was either hypoplastic or aplastic [5]. This additional knowledge has been widely used in previously reported literature and has helped in harvesting fibular-free flaps (FFFs) without donor/recipient complications. Previously, harvesting an FFF in the type IV variant where PrA is hypoplastic or aplastic used to result in donor’s foot ischemia or unusable fibular flap [6].

The detailed knowledge of the anatomy of termination of the PA and the variations of its bifurcation has gained importance for endovascular interventionists and plastic, vascular, and orthopedic surgeons, because of the upsurge in procedures like embolectomy, vascular grafting, FFF surgery, and high-tibial osteotomy, all of which require caution while approaching the popliteal region. Digital subtraction angiography (DSA) is considered the gold standard for evaluating the peripheral vascular system, and so is the case with the anatomy of the PA [1]. Only a few cadaveric and angiographic studies from India have reported variant anatomy of termination of PA, and none has been performed previously using a 128-slice computed tomography (CT). However, with progress in multidetector-row technology, computed tomography angiography (CTA) is now quite useful in evaluating the peripheral arterial system with high sensitivity and specificity [7].

This study aimed to measure the prevalence of variation in the termination of PA and its association with laterality and gender by using 128-slice CTA in a North-Indian population.

The abstract has been presented at the Summer Meeting of the Anatomical Society: Dublin 2022, and the abstract has been published in the Journal of Anatomy.

## Materials and methods

This was a retrospective study, conducted at King George’s Medical University, a tertiary care center in North India, in January-December 2021. Records of previously performed 128-slice CTA of patients (they might have undergone it due to any reason, other than patients with below-knee amputation, metallic implant in the leg region, total knee replacement, or complete thrombosis or atherosclerosis of artery) of age between five and 75 years, in the Department of Radiodiagnosis, were included. The multidetector-row technology CTA images of the lower extremities of these subjects were obtained and were reviewed by radiologists, anatomists, and cardiothoracic and vascular surgeons. Normally, the PA terminates into the anterior tibial artery and tibioperoneal trunk, at the lower border of the popliteus muscle. If the common trunk, following the origin of the first branch was 0.5 cm or less in length, it was considered trifurcation [1]. The branching pattern of the PA was observed and classified according to Kim’s classification, which is a modification of Lippert’s system [4,8]. The tibial plateau was taken as the reference point to define the high division of the PA. The attenuated artery was identified by diffuse narrowing with thin contrast. The prevalence of the variant pattern was calculated. The association between laterality and gender, with branching pattern was determined. The length of the tibioperoneal trunk was measured in type IA. Moreover, the association of the length of the tibioperoneal trunk was analyzed with gender and laterality.

The data were stored in Microsoft Excel (Microsoft Corporation, USA) and were analyzed using IBM SPSS Statistics for Windows, version 24.0 (released 2016, IBM Corp., Armonk, NY). Data were expressed as mean ± standard deviation and percentages. Normal descriptive analysis tests were used. P-values < 0.05 were considered significant. 

Approval for ethical waiver was obtained from the Institutional Ethical Committee of King George’s Medical University, Lucknow (ECR/262/Inst/UP/2013/RR-19).

## Results

**Figure 1:**
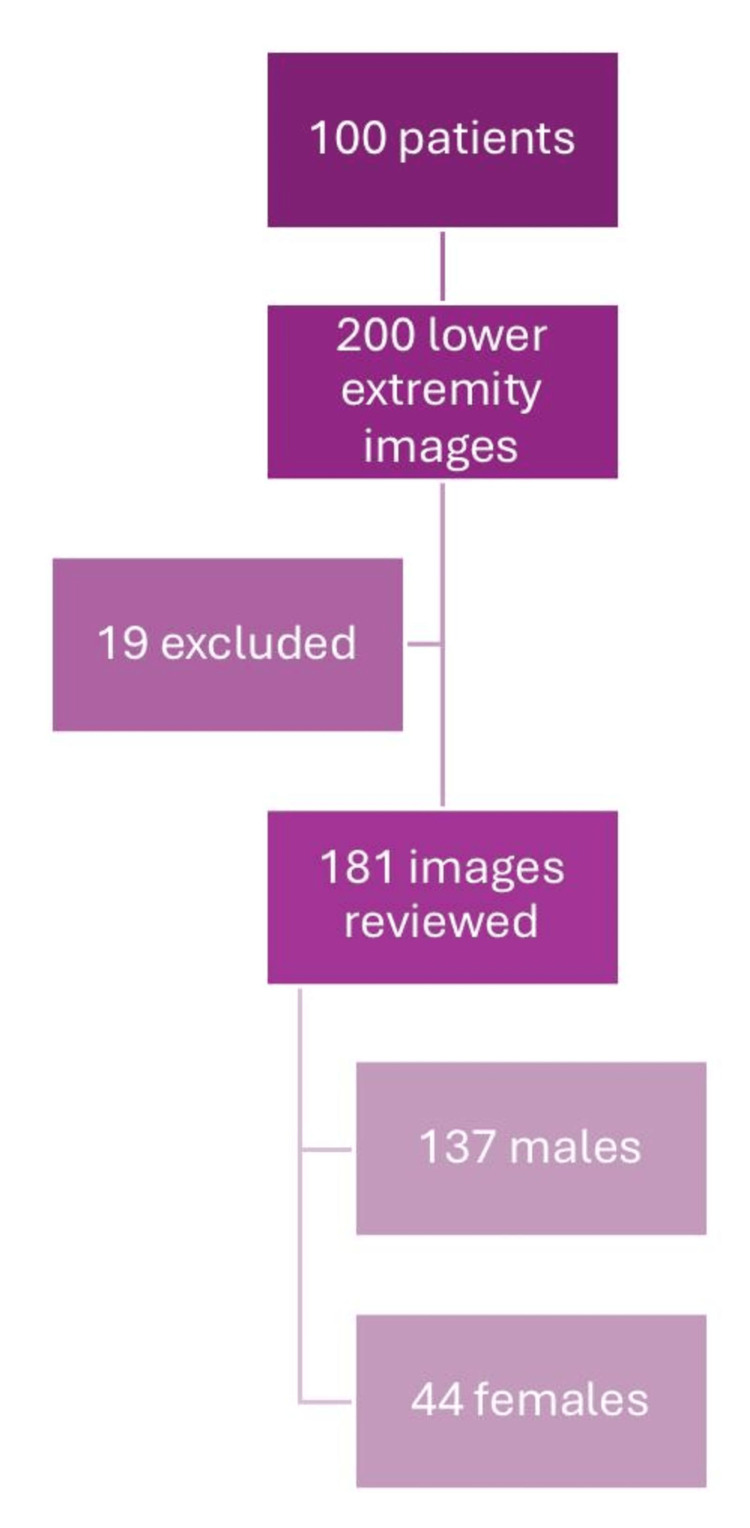
Flowchart of the study

One hundred patients were included in our study, from whom a total of 200 images were acquired. Out of the 200, 19 were excluded as they did not fulfill the study criteria - three were completely excluded (lower-limb images of both sides), and in 13, either-side image was excluded. Of the remaining 181, 137 were males and 44 were females (Figure 1).

**Figure 2:**
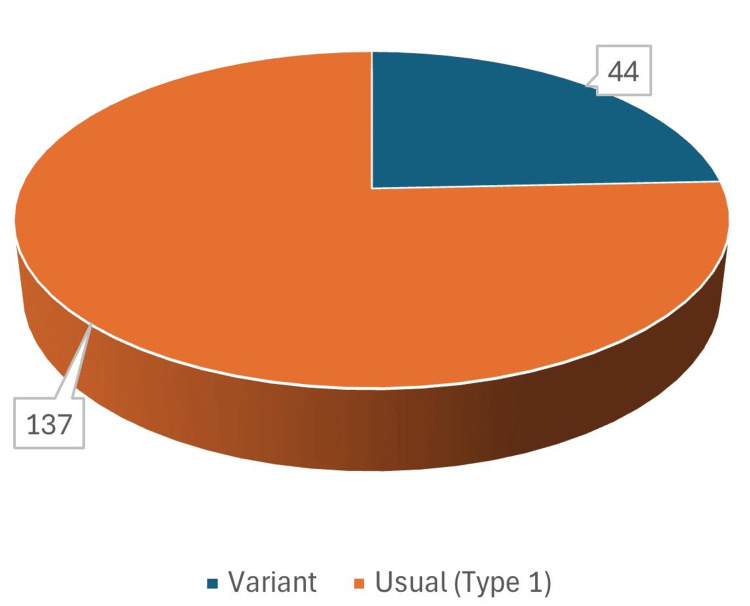
Variation in the termination pattern of the popliteal artery

No luminal content, wall thickening, or opacification was noted in any of the images. Variation in the termination pattern of the PA was observed in 44 (24.3%), whereas usual (type I) anatomy was noted in the rest (137) (Figure 2).

**Figure 3:**
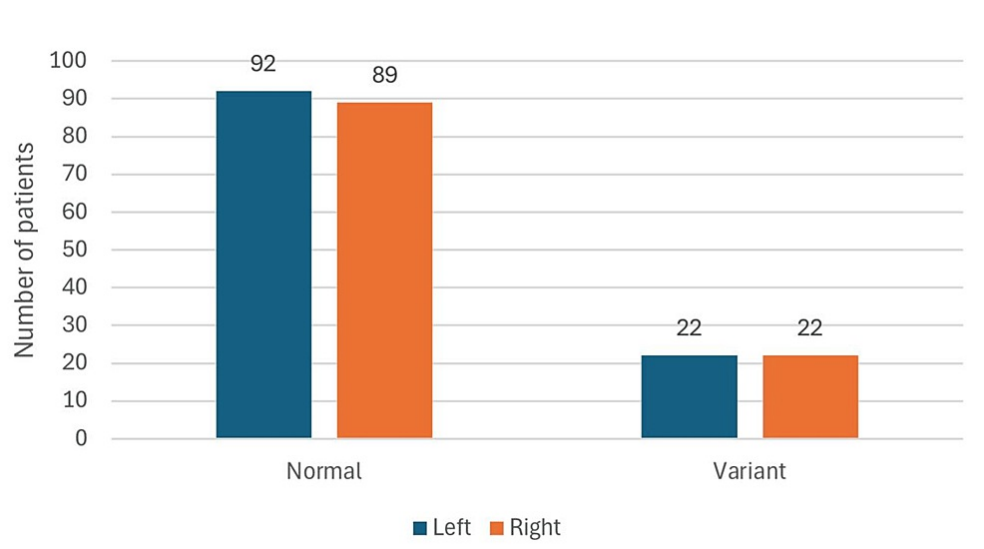
Prevalence of variant patterns on the right and left sides

The prevalence of variant patterns on the right and left sides is shown in Figure 3. The difference between the two sides was statistically insignificant.

**Figure 4:**
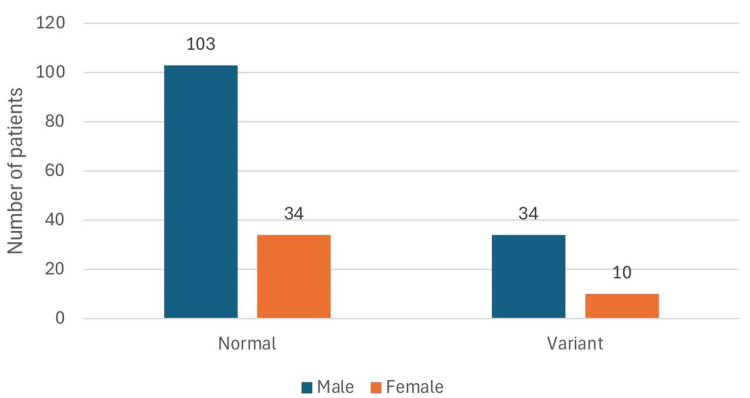
Prevalence of variant patterns in males and females

The prevalence of variant patterns in males and females is shown in Figure 4.

The difference in prevalence of variations in males and females was statistically insignificant. The prevalence of various termination patterns of the PA is shown in Table 1.

**Table 1:** Prevalence of various termination patterns of the popliteal artery. ATA: anterior tibial artery, ATPT: anterior tibioperoneal trunk, PTA: posterior tibial artery, PrA: peroneal artery

Type	Termination pattern		Number of patients (percentage)
TypeI	Normal level of termination		142 (78.45)
IA	Usual pattern	137 (75.69)	
IB	Trifurcation	4 (2.21)	
IC	Anterior tibioperoneal trunk (ATPT)	1 (0.56)	
TypeII	High division of the popliteal artery		4 (2.21)
IIA1	ATA is the first branch and has a normal course	3 (1.66)	
IIA2	ATA is the first branch and has a medial initial curve	1(0.56)	
IIB	PTA is the first branch, the common trunk for PrA and ATA (ATPT)	0	
IIC	PrA is the first branch, common trunk for the ATA and PTA	0	
Type III	Hypoplastic/aplastic terminal branches		35 (19.34)
IIIA	Hypoplastic/ aplastic PTA with distal substitution of the PTA by the PrA	20 (11.05%)	
IIIB	Hypoplastic/aplastic ATA with distal Substitution of the dorsalis pedis artery by PrA	9 (4.97%)	
IIIC	Hypoplastic/aplastic PTA and ATA with distal substitution of the PTA and DPA by PrA	3 (1.66%)	
IVA	Aplastic PrA	3 (1.66%)	

**Figure 5:**
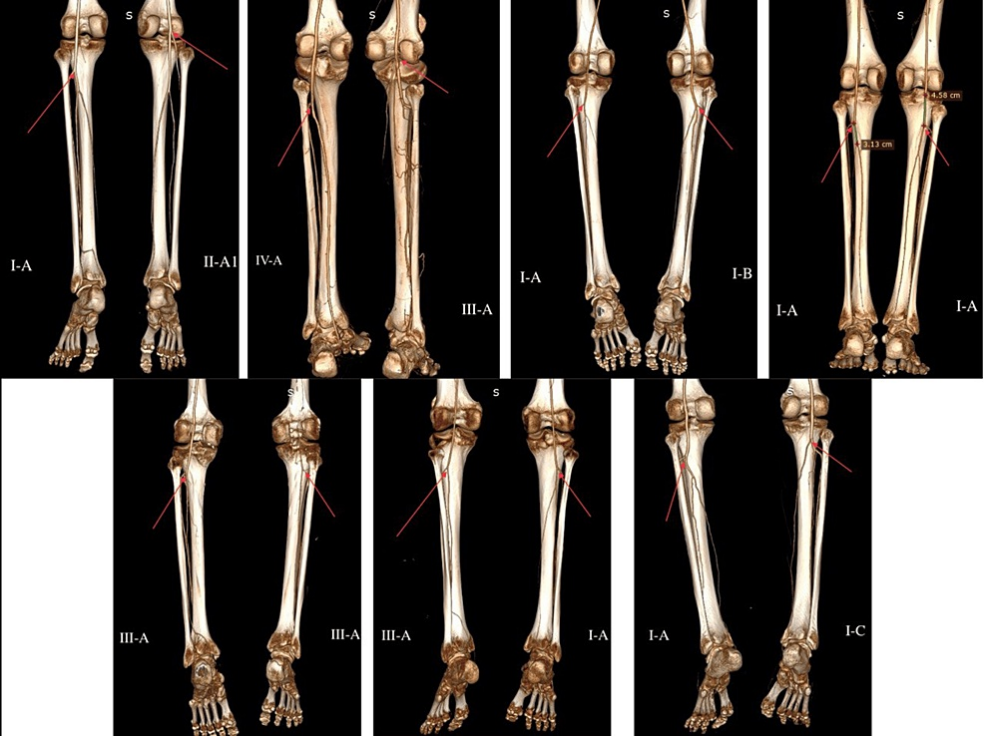
3D reconstruction images of CT angiography of the terminal portion of the popliteal artery in patients showing the spectrum of the variant anatomy. Termination of the popliteal artery is shown by red arrows.

The most frequently observed variation was type IIIA, followed by type IIIB. Types IIB and IIC were not observed in our study (Figure 5).

The comparison of variant branching patterns in the respective limbs in the bilaterally evaluated patients (84) is summarized in Table 2.

**Table 2:** Comparison of variant branching patterns in the respective limbs in the bilaterally evaluated patients B/L: bilateral, U/L: unilateral

	Type IA		Variant pattern		Total
	B/L	U/L	B/L Similar	B/L Different	
No. of cases	55 (65.48%)	16 (19.05%)	7 (8.33%)	6 (7.14%)	84 (100%)

Type IIIA was bilaterally observed in six out of seven cases.

The comparison of the mean length of TPT in the Type IA pattern, between the two sides, and between the two sexes is summarized in Table 3.

**Table 3:** Comparison of the mean length of TPT in the type IA pattern, between the two sides, and between the two sexes TPT: tibioperoneal trunk

	Right	Left	Male	Female	Total
Mean Length of TPT (cm) in type IA	3.21 ± 1.02	2.82 ± 0.93	2.9 ± 1.00	3.37 ± 0.85	3.00 ± 0.99
P-value	0.016		0.014		

The difference between the sides and sexes was statistically significant.

## Discussion

The comparison of the findings of various MDCT angiographic studies in PA variations, including results from our studies, is summarized in Table 4.

**Table 4:** Literature review TPT: tibioperoneal trunk

Authors	Year	Limbs	Prevalence of variation	Most common variant	Mean TPT length	
Kil et al. [9]	2009	1242	10.8%	IIIA (5.1%)	-	
Yanik et al. [10]	2015	126	16.4%	IC, IIA2 (4.4%)	-	
Calisir et al. [11]	2015	636	13%	IB (4.2%)	-	
Oztekin et al. [4]	2015	495	12.5%	IIIA (3.9%)	2.96 cm	
Demirtas et al. [6]	2016	1261	11.3%	IIIA (3.5%)	-	
Markande et al. [12]	2019	168	9.44%	IIA1,2; IIIB (2.36%)	-	
Oner et al. [1]	2020	340	10.6%	IB (3.2%)	Yes	
Our study	2022	181	24.3%	IIIA (11.05%)	3.00 ± 0.99 cm	

The prevalence of variations in the termination pattern was found to be higher in our study, as compared to the other studies reported in the literature, which was 24.3%. It is much higher than the previous Indian study conducted by Markande et al., which reported a prevalence of 9.44% variation [12]. The probable reason behind this may be the methodological and regional differences.

The most common variant of PA reported in previous literature is type I, whose prevalence varies between 89% and 96%, with the most common being type IA [2,9,13,14,15,16]. Our findings are similar to these data, with the prevalence of type I and type IA being 78.45% and 75.69%, respectively.

The reported incidence for the type II branching pattern ranges between 1.6% and 7.8% [2,4,9,10,11,14], but this pattern had a relatively low incidence (2.21%) in the present study.

Type IIIA was the most common variant in our study (11.05%), as well as in two others by Demirtas et al. and Oztekin et al., which were 3.5% and 3.9%, respectively [7,17]. The probable reason for the difference in frequency can be attributed to the differences in the sample size and racial variations.

In the present study, 1.66% of the cases showed a type IV-A variant. Similar observations were reported by Blackwell et al., Manaster et al., and Sadhu et al. [18,19,20]. Variations were more common in females in the study conducted by Oner et al. [1], but the difference in genders was not significant in our study.

The mean TPT length in the studies by Oner et al. and Ongsiriporn et al. were nearly similar, including this study [1,5]. In our study, the mean length of TPT in females was significantly higher than in males, whereas Oner et al. [1] did not find any such association. The mean length on the right side was significantly higher than that on the left, in both our study and in that conducted by Oner et al. [1]. The variation can be explained based on embryological basis. It occurs due to the combination of persistent primitive arterial segments, abnormal fusions, or segmental hypoplasia or absence [10].

Limitations

First, this is a retrospective study, and there are chances of limitations to the quality and completeness of the existing CTA images and associated clinical data in the departmental records. Second, a sample size of 100 patients may not fully represent the wider population. The gender distribution of 137 males and 44 females could involve gender bias. Third, the study is based on a population from northern India, which might raise questions about the generalizability of the results to other ethnic or geographic populations, due to possible racial and regional variations. Fourth, the large age group includes both pediatric and geriatric patients, whose vascular anatomy might vary from the adults, which might have an effect on the observed patterns and their clinical implications. Fifth, all cases did not have bilateral representation, which may limit the analysis of symmetry in the variation patterns. Moreover, the research could not find certain variations (types II-B and II-C). Lastly, the research mainly aims to study the anatomical variations without much clinical correlation that these variations might bear on surgical procedures and their outcome factors.

## Conclusions

This study highlights the significant variability in the termination patterns of the PA, with a notable 24.31% of cases showing variant branching patterns. The predominance of type III variants, particularly type III-A, shows the requirement for meticulous preoperative planning and an intricate understanding of the anatomy of surgeons performing procedures in the lower limb. The observed differences in the lengths of the tibial-peroneal trunk between sexes and sides further underscore the importance of individualized assessment. The use of 128-slice CTA has given intricate information, which can improve the precision of vascular and orthopedic surgeries, in a way targeting to lessen the postoperative complications and improve patient outcomes. The amalgamation of comprehensive knowledge of anatomy into surgical practice is mandatory for enhancing surgical accuracy and patient care.
